# Body Composition, Eating Habits, and Disordered Eating Behaviors among Adolescent Classical Ballet Dancers and Controls

**DOI:** 10.3390/children10020379

**Published:** 2023-02-15

**Authors:** Panagiota Chaikali, Ioanna Kontele, Maria G. Grammatikopoulou, Eleftheria Oikonomou, Theodoros N. Sergentanis, Tonia Vassilakou

**Affiliations:** 1 Department of Public Health Policy, School of Public Health, University of West Attica, 196 Alexandras Avenue, GR-11521 Athens, Greece; 2 Department of Rheumatology and Clinical Immunology, Faculty of Medicine, School of Health Sciences, University of Thessaly, Biopolis, GR-41110 Larissa, Greece; 3 Independent Researcher, GR-16674 Glyfada, Greece

**Keywords:** adolescence, ballet dance, diet, nutrition, eating disorders, disordered eating behaviors, weight, fat mass, relative energy deficiency in sport (RED-S), athlete

## Abstract

Adolescent classical ballet dancers are nutritionally vulnerable, as they try to retain a lean body shape during a life period of high nutritional requirements due to rapid growth. Studies conducted on adult dancers have indicated a high risk for the development of disordered eating behaviors (DEBs), but research on adolescent dancers remains scarce. The aim of the present case-control study was to compare the body composition, dietary habits, and DEBs of female adolescent classical ballet dancers and their non-dancer same-sex peers. Self-reported questionnaires, namely the Eating Attitudes Test-26 (EAT-26) and a 19-item Food Frequency Questionnaire (FFQ), were used for the assessment of habitual diet and DEBs. The assessment of body composition included the measurements of body weight, height, body circumference, and skinfolds and bioelectrical impedance analysis (BIA). The results indicate that the dancers were leaner than the controls, with lower weight, BMIs, and hip and arm circumferences, leaner skinfolds, and less fat mass. No differences were observed between the two groups regarding eating habits and the EAT-26 scores, but almost 1 out of 4 (23.3%) participants scored ≥ 20, indicative of DEBs. Participants with an EAT-26 score ≥ 20 had significantly higher body weight, BMIs, body circumferences, fat mass, and fat-free mass than those with a score < 20. Adolescents must be educated on nutrition and healthy methods to control body weight through evidence-based information and programs, and whenever appropriate, also through individual counseling by the appropriate health professionals.

## 1. Introduction

During adolescence, rapid physical and psycho-emotional changes occur, and adolescents face a number of challenges, including school demands, social relationships with peers and family, and a growing need for autonomy [1,2]. Nutritional demands are very high during this period, and adolescents are a nutritionally vulnerable group [3]. In Europe, 1 in 5 adolescents is either overweight or obese [4], and less than half of teens reported healthy eating habits, including eating breakfast and consuming fruits and vegetables on a daily basis [5]. On the other hand, many adolescents are often dissatisfied with their body weight and shape; they consider themselves fat, even if they are not, and they often use unhealthy weight loss methods [5,6,7].

Sports participation during adolescence is considered necessary for proper development and the prevention of chronic diseases [1], although there are some sports that have great demands for a lean shape or a low body weight [8]. Classical ballet is a demanding activity requiring great physical skill, flexibility, and overall strength, and a lean shape is considered desirable, mainly for aesthetic reasons [9]. Low body weight is associated with performance benefits, as it makes it easier to balance en pointe and gives an advantage to the male partner to lift the female dancer more easily [10]. The research indicates that ballet dancers have a lower body weight, body mass index (BMI), and smaller body circumference than non-dancers [11,12,13,14].

Attaining the ideal body weight and shape could be challenging for many female adolescent dancers, especially for those aiming to become professional dancers, as they struggle to retain a low body weight during a period of accelerated body growth [15]. Therefore, dancers may use unhealthy methods to reduce or retain body weight, including energy-restrictive diets, skipping meals, vomiting, using laxatives, or dehydration techniques, all being indicative of disordered eating behaviors (DEBs) [16,17]. These methods may affect the normal development of adolescents and result in serious nutritional risks, such as deficiencies in macronutrients and micronutrients, dehydration, electrolyte imbalances, irregularities in menstruation, and low bone density [18]. More serious eating disorders could last even through adult life [19]. Professional dancers are considered to be at great risk for developing relative energy deficiency in sport (RED-S), which includes impairments in many physiological functions, such as menstrual and cardiovascular function, growth, metabolic rate, bone health, immunity, and many others. RED-S is caused by the low energy availability that occurs when an athlete under-consumes energy in the diet, resulting in a failure to cover the energy demands of growth and exercise and support bodily functions for optimal health and performance [20]. Pressure for attaining a low body weight or a lean shape that comes from the sports environment may further increase the risk of developing DEBs [21,22,23]. In addition, body dissatisfaction and perfectionism are some of the strongest risk factors for developing eating disorders during adolescence [24].

Recent studies have indicated a greater prevalence of eating pathology in ballet dancers when compared to controls, with a lifetime prevalence of any eating disorder reaching 50% in professional dancers [17,25]. Moreover, studies have shown that dancers’ macronutrient and micronutrient intake is lower than recommended [11,14,26]. Although research conducted on adolescent dancers remains scarce, a lower prevalence of eating disorders has been reported in comparison to that of adult dancers [14,27,28,29], possibly explained by the commonly non-professional practice of dancing taking place during adolescence. In Greece, few studies have examined anthropometric characteristics, eating habits, and disordered eating among dancers, mainly in professional adult dancers [11,30,31]. Nevertheless, no study has compared the anthropometric characteristics, dietary habits, and DEBs of adolescent non-professional dancers and their non-dancer peers.

Therefore, the purpose of the current case-control study was to evaluate the body composition, eating habits, and DEBs in a sample of female non-professional adolescent ballet dancers and examine possible differences compared to a control group of non-dancer adolescent girls. It was hypothesized that the dancers would have lower weight, BMI, and fat mass than their counterparts, but they would also have more DEBs because of the pressures they face for attaining a low body weight and a lean shape.

## 2. Materials and Methods

### 2.1. Participants

The present case-control study included 90 adolescent girls in total, all junior and senior high school students. The case group (*n* = 46) included dancers from an amateur classical dance school situated in metropolitan Athens, Greece. The control group (*n* = 44) included girls from public junior high and senior high schools located in the prefecture of Attica. The inclusion criteria for the participants were the following: (a) being female, (b) between 11 and 17 years old, (c) providing personal and parental/guardian consent, (d) able to complete the questionnaires, and (e) a student at an amateur classical dance school (for the dancer group). The exclusion criteria were (a) younger than 11 or older than 17 years, (b) not providing personal and parental consent, (c) unable to provide information (i.e., complete the study’s questionnaires), or (d) having a nutrition-related problem or diagnosis (e.g., diabetes mellitus).

### 2.2. Ethical Permission, Consent, and Anonymity

Approval for the current study was granted by the Greek Ministry of Education and Religious Affairs (number of approval 126463/Γ2/14-10-2009) after the positive opinion of the Institute of Educational Policy. Every adolescent participated in the study only after the informed written consent of a parent or legal guardian was granted. All girls were also asked to provide their personal written consent. Participation in the study was anonymous and voluntary. The principal investigator (P.C.) explained to the participants that they retained the right to withdraw at any time.

### 2.3. Tools

Structured questionnaires were provided to all participants for completion during school time in the presence of the principal investigator and the school teacher. The first part of the questionnaires included questions on the general characteristics of the girls (age and social characteristics), some questions regarding eating habits (daily frequency of eating occasions, consumption of breakfast, use of dietary supplements, dieting), body image characteristics (weighting, body image), physical activity patterns, smoking, alcohol consumption, and data regarding menstruation.

#### 2.3.1. Dietary Habits

A 19-item Food Frequency Questionnaire (FFQ) was used to collect data regarding the frequency of consumption of specific food groups. The food groups were selected based on the groups that are described in the Greek dietary guidelines [32] and the participants were asked to indicate the daily, weekly, or monthly frequency of the consumption of each food group (4–6 times/day, 1–3 times/day, 5–6 times/week, 2–4 times/week, 1 time/week, 1–3 times/month, a few times or never). Consumption frequencies were compared to the Greek Dietary Guidelines for children and adolescents [33].

#### 2.3.2. Disordered Eating Behaviors (DEBs)

DEBs were assessed with the use of the Eating Attitudes Test (EAT-26) by Garner et al. [34]. EAT-26 is a 26-item self-administered questionnaire. The answers are scored from 0 to 3 as follows: sometimes/rarely/never = 0, always = 1, usually = 2, often = 3, with the last question receiving a reversed score. The total score ranges from 0 to 78, and a score of ≥20 is indicative of DEBs. Three sub-scale scores are calculated, namely (i) Dieting, (ii) Bulimia and Food Preoccupation, and (iii) Oral Control. The EAT-26 questionnaire is not used in order to diagnose eating disorders but only as a screening tool that can detect possible cases of eating disorders, and those with an indicative score should seek further evaluation from health experts. The questionnaire has been previously translated and validated in the Greek population [35]. In the present study, the Cronbach α for the EAT-26 was calculated at 0.79.

#### 2.3.3. Dance-Related Information

Finally, the dancer group completed an additional questionnaire regarding the total dancing years, the frequency and duration of the training sessions, and participant perceptions regarding the ideal body type for a dancer. Furthermore, the girls were asked whether they had received diet advice from others and what their aim was regarding the pursuit of a professional dance career.

### 2.4. Body Composition Indices

Selected body composition measurements were implemented by the principal investigator during other school hours without the presence of the school teacher. These included body height, weight, circumference, skinfold thickness, and body composition assessment using bioelectrical impedance analysis (BIA).

The participants’ heights were measured in bare feet to the nearest 0.5 cm with a portable stadiometer (Seca, Hamburg, Germany). Their weights were measured to the nearest 0.1 kg with a portable digital weight scale. The BMI was calculated for all participants as their body weight (kg) divided by their height (m^2^). The participants were classified as underweight, normal weight, or overweight and obese, according to their BMI, using the International Obesity Task Force BMI cut-offs for children and adolescents, linking BMI values at 18 years (16, 17, 18.5, 25, and 30 kg/m^2^) to the child and adolescent centiles [36].

Body circumferences were measured to the nearest 0.1 cm with the use of a common anelastic measuring tape and included the measurements of the waist, hip, and upper arm circumferences. During the measurements, the participants stood in an upright position with their feet together for all measurements. The waist circumference was measured as the horizontal line at the high point of the iliac crest, the hip circumference was measured at the maximum extension of the buttocks, and the upper arm circumference was measured as the circumference midway between the tip of the shoulder and the tip of the elbow.

Biceps, triceps, subscapular, and suprailiac skinfolds were measured with a Slimguide skinfold caliper on the right side of the person, and the measurements were recorded in the nearest millimeter. Each measurement was performed twice, and the median value was recorded for every participant. In order to calculate the total fat mass (FM), the body density was firstly calculated with the Brook equation, as follows: Body Density (g/cm^3^) = 1.2063 − 0.0999 × log sum of 4 skinfolds [37]. Then, the body fat percentage of body weight was calculated using the Siri equation as Body Fat (% of body weight) = (495/Body Density) − 450 [38].

Finally, bioelectrical impedance analysis (BIA) was performed with a portable RJL model 101 body composition analyzer (RJL—Systems, Mt. Clemens, MI, USA). The analyzer estimated a resistance value that was used to calculate the fat-free mass (FFM) and FM. For the calculation of the FFM, the Houtkooper equation for adolescents was used, as follows: FFM = 1.31 + (0.61 × height [cm]^2^/resistance) + (0.25 × body weight [kg]) [39]. Then, the FM was calculated as FM (kg) = body weight (kg) − FFM (kg).

### 2.5. Statistical Analyses

Statistical analyses were performed using the Statistical Packages for Social Sciences (SPSS) version 22.0 (SPSS Inc., Chicago, IL, USA). For all analyses, the level of statistical significance was set at 0.05. Continuous variables were checked for normality, and normally distributed data are presented as means with standard deviation, while skewed data are presented as medians with their respective interquartile range (IQR). Categorical variables are presented as frequencies and percentages.

Chi-squared tests were performed in order to examine the differences between the two groups regarding their eating habits, DEBs, and body dissatisfaction characteristics.

Independent samples t-tests and Mann–Whitney tests were used to examine the differences between the dancers and controls regarding the anthropometric characteristics and their scores in the EAT-26. Moreover, independent sample t-tests were used in order to examine the differences in BMI and fat mass between the participants that followed specific eating habits or not, as well as between participants with scores under or ≥20 in the EAT-26. Finally, the Spearman rho correlation coefficient was used for investigating the relationships between the continuous variables.

## 3. Results

### 3.1. Anthropometric Characteristics

A total of 90 adolescent girls completed the questionnaires and the body measurements, with 46 of them being dancers, and the rest of them in the control group. The majority of the participants’ parents (70.0% of mothers and 68.9% of fathers) had tertiary education, with no significant differences between the dancers and controls. The mean age of the participants was 13.7 (1.3) years, with no significant difference between the two groups.

As shown in Table 1, significant differences were found between the dancers and controls regarding all anthropometric characteristics, except for height, waist circumference, and FFM. The dancers exhibited lower body weight, hip circumference, and skinfold measurements compared to the controls. Accordingly, the dancers had a lower FM than the controls, although the FFM was similar between the two groups. Similarly, the dancers had lower BMIs and a smaller proportion was overweight compared to the controls.

### 3.2. Eating Habits

The eating habits of the participants are shown in Table 2. Despite numerical differences in the percentages, the differences between the two groups did not reach statistical significance.

The BMIs and FM of the participants were compared according to their specific eating habits (Table 3). It was found that the participants who consumed more than three meals/snacks per day had lower BMIs (*p* = 0.028) than those who consumed fewer meals per day, while those who consumed breakfast every day had lower BMIs (*p* = 0.042) and lower FM (*p* = 0.020) than those who did not eat breakfast every day. Moreover, participants who consumed nuts at least twice per week had lower BMIs (*p* = 0.001) and lower fat mass (*p* = 0.001) than those who ate nuts less often.

### 3.3. DEB and Body Dissatisfaction

As indicated in Table 4, 21 participants (23.3%) scored ≥ 20 in the EAT-26, which is indicative of DEBs. No differences were observed between the dancers and controls, as almost the same percentage of girls within each group scored 20 or more. Accordingly, no differences were found between the two groups regarding the scores in the EAT-26 and its three subscales.

It was also found that the participants who scored ≥ 20 in the EAT-26 had a significantly higher body weight (*p* = 0.032), BMI (*p* = 0.014), waist (*p* = 0.022) and hip (*p* = 0.023) circumferences, FM (*p* = 0.033), and FFM (*p* = 0.008) than those who scored < 20 (Table 5).

Moreover, positive correlations were found between the EAT-26 score and weight, hip circumference, and fat mass (*p* < 0.05) according to the Spearman correlation.

Additionally, a significant number of participants reported DEBs and body dissatisfaction, as indicated in Table 6. More than 3 out of 10 adolescent girls mentioned that they had followed a slimming diet at some time in their life, while 11.2% were on a diet during the study. Furthermore, 31.1% of participants reported that they had followed a slimming diet that was not prescribed by a health professional. Only three participants mentioned having used vomiting or laxatives in order to control their weight, but all of them were dancers. Moreover, 28.9% of the participants reported frequent weighing. No significant differences were found between the dancers and controls regarding all of the above-mentioned behaviors. Regarding body dissatisfaction, almost half of the participants were not satisfied with their bodies, and 1 in 4 reported feeling normal but wanting to weigh less. No statistically significant differences between the dancers and controls were found. Finally, regarding the questions that were addressed only to the dancers, 4 in 10 stated that they felt their body was not ideal for a dancer, and 17.4% had lost weight in order to participate in dancing exams or performances. Moreover, 13.0% of the dancers reported having received diet advice from their coaches, 8.7% from their teammates, and 26.1% from their parents.

## 4. Discussion

### 4.1. Body Composition

According to the findings of the study, the dancers had significantly lower body weight, BMIs, hip and arm circumferences, skinfolds, and fat mass than the controls. Similarly, Stokic et al. (2005), Toro et al. (2009), and Yang et al. (2010) have shown that adolescent ballet dancers had significantly lower values of body weight, BMI, and fat mass compared to controls [12,40,41]. Similar results have also been observed among adult dancers [11,13]. Additionally, the mean BMI of dancers in the current study was within the normal range, and the majority (78.3%) had a normal weight, as has also been indicated by Rosselli et al. (2022) and Beck et al. (2015) in groups of adolescent dancers [27,42]. Moreover, it was found that significantly more controls were overweight than the dancers, but underweight prevalence was similar between the two groups. Contrary to this finding, Castelo-Branco et al. (2006) found a higher prevalence of underweight in a group of adolescent dancers compared to controls [14]. Nevertheless, it should be noted that ballet is an activity that demands a lean shape, and it could be hypothesized that overweight girls could be less likely to engage in ballet dancing.

### 4.2. Dietary Habits and Correlations with Anthropometric Characteristics

Regarding dietary habits, no difference was found between the dancers and the controls. Even though more dancers than controls tended to eat breakfast, fruits, vegetables, and dairy on a daily basis, the differences were not statistically significant. In the total sample, only 3 out of 10 participants had more than 3 meals or snacks per day, and 67.8% reported daily breakfast consumption. Accordingly, in the Health Behaviour in School-aged Children (HBSC) study, 65.4% of adolescent girls reported daily breakfast consumption [43], while in a recent study on Greek adolescent gymnasts, 72.5% had breakfast every day [44]. Regarding the food groups, 56.7% of the participants reported daily fruit consumption, and 36.7% reported daily vegetable consumption. This finding is similar to the results of a study on adolescent dancers in Poland that observed daily consumption of fruits and vegetables in 49% and 36.9% of the dancers, respectively [45]. In comparison to the recent HBSC study conducted on Greek adolescents, a greater proportion of adolescents in the current study appeared to consume fruits daily, while the consumption of vegetables was similar [5]. Moreover, a significant proportion of adolescents in the current study did not appear to follow the national dietary guidelines for daily or weekly consumption of specific food groups. More specifically, only 63.3% of the participants reported daily consumption of dairy, and 50.0% reported daily consumption of cereal and bread. Accordingly, most of the participants did not follow the guidelines for the consumption of fish, nuts, eggs, and legumes twice per week. On the other hand, almost half of the participants reported the consumption of red meat less than twice per week, as it is suggested in the guidelines. These findings agree with recent studies that have shown that adolescents in Greece do not follow the national dietary guidelines [43,46].

Regarding the associations between the participants’ specific eating habits and weight status, significant findings were revealed regarding the number of eating occasions and breakfast and nut consumption. Specifically, it was found that the participants who consumed more than three meals daily had significantly lower BMIs than the participants who had fewer meals per day. This finding is similar to the findings of Ritchie et al. (2012), who indicated that a lower eating frequency may predict a greater increase in adiposity in adolescent females [47]. Moreover, the meta-analysis by Kaisari et al. (2013) showed that a higher eating frequency was associated with a lower body weight status in children and adolescents [48]. However, it should be noted that only 33.3% of the participants reported having more than three eating occasions daily, which is lower than the findings of Szczepańska et al. (2021) regarding adolescent dancers [45]. Additionally, it was found that the participants who consumed breakfast on a daily basis had lower BMIs and FM, compared to those who did not eat breakfast daily. This finding is in agreement with a great number of studies that have shown a protective role of breakfast consumption against overweight in children and adolescents [49,50,51].

An interesting finding of the current study was that participants who ate nuts at least twice a week had significantly lower BMIs and fat mass than those who did not consume nuts so frequently. This finding agrees with the results of other studies conducted among adolescents in the community, as well as adolescent athletes [44,52]. Accordingly, an international study with participants from 35 countries revealed that adolescents who consumed nuts several times per week exhibited a lower BMI when compared to those who consumed nuts rarely or never, possibly due to the fact that nuts are good sources of nutrients that may promote satiety (proteins, fatty acids, and fibers) [53].

### 4.3. DEBs and Body Dissatisfaction

Almost one in four (23.3%) participants in the present study scored 20 or more in EAT-26, indicating DEBs. This finding is in accordance with the findings of other studies in Greece that have shown a prevalence of DEBs in adolescent girls of 20% to 25% [54,55,56]. This study was the first in Greece that examined DEBs in adolescent dancers, although the prevalence of 23.9% that was found in the dancer group is lower than the prevalence that was found by Yannakoulia et al. in adult dancers [11] and that in the study by Theodorakou and Donti (2013) in female aesthetic athletes [57], but it is higher than the findings of Kalyva et al. (2021) regarding adult dancers [30].

Accordingly, 3 out of 10 participants reported having followed a slimming diet at any time, while 11.2% were on a diet during the period of the study, and 31.1% mentioned having followed a diet that was not prescribed by a health professional. According to the findings of the HBSC survey, 30% of 15-year-old girls and 24% of 13-year-old girls in Greece engaged in weight reduction behaviors, while the corresponding percentages in the European region of the World Health Organization were 26% and 21%, respectively [58]. Accordingly, in a study on Greek adolescents, 32.8% reported that they were on a weight loss diet to lose or maintain weight in the last month [59].

It should also be noted that almost half of the participants were not satisfied with their body image, and 1 in 4 reported feeling normal but wanting to weigh less. According to the literature, dissatisfaction with weight and body image increases from early to late adolescence and is often associated with increased weight, real or perceived [60]. Moreover, the HBSC survey findings indicated that 33% of 13-year-old and 36% of 15-year-old girls think they are too fat [5], while Chaikali et al. found that 22.6% of Greek adolescent girls with normal weight considered that they were overweight [59]. Nevertheless, studies among dancers have indicated that, even though they have a normal body weight (or even a low body weight), many dancers are dissatisfied with their body weight and body shape and want to be thinner [61].

Finally, in the current study, no differences were found between the dancers and controls regarding the EAT-26 score, other DEBs, or body dissatisfaction. These findings are in contrast to the findings of other studies in adolescents and adult dancers that have shown a higher prevalence of eating pathology in dancers versus controls [14,17]. On the other hand, a significant number of studies have indicated that adolescent and young ballet dancers do not have a higher risk of developing eating disorders compared to controls [27,40,62]. This could be explained by the fact that dancing at younger ages (especially under 14 years old) is not professional, and therefore, pressures to be thin may be lower.

### 4.4. Correlation between DEBs and Anthropometric Characteristics

The present study revealed a positive association between the EAT-26 scores and anthropometric characteristics. The participants with DEBs seemed to be heavier than the participants that had low scores in the EAT-26. Similar findings have been observed in studies on adolescents of the general population, where overweight adolescents are more likely to engage in dieting and have a greater risk of disordered eating symptomatology than their normal-weight peers [63,64].

A positive relationship between DEB symptoms and BMI has also been found in studies on dancers [30,62,65,66]. Specifically, Kalyva et al. (2021) showed that the higher the BMI, the higher the eating self-control and perceived pressure from other significant persons [30]. On the other hand, a study in Brazil found that dancers who had a percentage of body fat above or below what is considered normal for the profession presented a higher risk for eating disorders [66].

### 4.5. Limitations of the Study

The current study has some limitations that should be discussed. First, this was a cross-sectional study; therefore, it was not possible to demonstrate a causal relationship between the variables studied. Second, the questionnaires that were used were self-reported. So, there is a possibility that some participants reported eating habits and behaviors that did not actually describe their real habits. In order to ensure that the participants completed the questionnaires as unaffectedly as possible, anonymity was ensured. Furthermore, in this study, no eating disorder diagnostic assessment was performed, though the EAT-26 is a commonly used screening tool for disordered eating symptomatology. An additional limitation comes from the fact that the sample size was relatively small, which did not allow for the conduction of more complex regression models. Finally, it should be noted that only female participants were included in the study. This was decided based on the fact that, in Greece, the number of males that participate in ballet dance classes is limited.

### 4.6. Strengths of the Study

To our best knowledge, the present study is the first conducted in Greece to examine body composition, eating habits, and DEBs in adolescent non-professional dancers. Moreover, the study included body composition measurements with BIA, skinfolds, and circumferences, which makes the results of the anthropometric characteristics more reliable than the self-reported characteristics.

## 5. Conclusions

The current study examined the body compositions, eating habits, and DEBs of female non-professional adolescent dancers and adolescent non-dancer girls. The dancers were leaner than the controls, although they did not present higher eating disorder scores. The dancers and controls did not differ significantly regarding their eating habits, body dissatisfaction, or DEBs. However, a significant percentage of the total sample presented with body dissatisfaction and DEBs, while girls with high disordered eating scores had higher BMIs, FM, and FFM than those with lower scores.

Further elaborating on these findings, we could suggest that adolescent non-professional female dancers do not have a greater risk of developing eating disorders than their peers, although all adolescents could be considered a high-risk group. Moreover, a significant percentage of all adolescents did not follow the guidelines for healthy nutrition. Therefore, adolescents should become a population of priority for interventions regarding healthy eating, as well as for the prevention and control of DEBs and eating disorders. More studies on this vulnerable population are needed in order to examine the risk factors that contribute to the development of eating disorders.

## Figures and Tables

**Table 1:** Participant ages and anthropometric characteristics.

	Total (*n* = 90)	Dancer Group (*n* = 46)	Control Group (*n* = 44)	*p*
Age (years)	13.7 (1.3)	13.4 (1.5)	14.0 (1.1)	0.072
Height (cm)	160.8 (6.0)	160.8 (6.5)	160.8 (5.6)	0.969
Body weight (kg)	54.8 (10.4)	52.0 (8.2)	57.8 (11.6)	0.008
BMI (kg/m^2^)	21.1 (3.6)	20.0 (2.5)	22.3 (4.3)	0.004
Waist circumference (cm)	68.0 (64.0, 73.5)	67.5 (64.0, 71,0)	70.0 (64.5, 75.5)	0.137
Hip circumference (cm)	94.6 (8.8)	91.3 (7.4)	98.0 (8.9)	0.000
Upper arm circumference (cm)	24.0 (23.0, 27.0)	24.0 (22.5, 25.0)	25.5 (23.0, 29.0)	0.014
Triceps skinfold (mm)	14.0 (4.1)	12.8 (3.4)	15.3 (4.4)	0.004
Biceps skinfold (mm)	7.2 (2.9)	6.30 (2.0)	8.3 (3.4)	0.001
Subscapular skinfold (mm)	10.5 (8.0, 14.0)	9.6 (7.0, 12.0)	12.7 (9.2, 16.1)	0.003
Suprailiac skinfold (mm)	13.0 (5.3)	11.5 (4.7)	14.5 (5.5)	0.007
Fat mass (kg)—Skinfolds	13.5 (6.4)	11.4 (4.7)	15.6 (7.2)	0.002
Fat mass (kg)—BIA	14.4 (6.3)	11.9 (4.6)	17.2 (6.8)	0.000
Fat mass (%)—Skinfolds	23.5 (7.2)	21.3 (6.3)	25.9 (7.4)	0.002
Fat mass (%)—BIA	25.5 (6.9)	22.5 (5.9)	28.8 (6.5)	0.000
Fat free mass (kg)—BIA	40.2 (5.3)	40.0 (5.3)	40.4 (5.5)	0.725
BMI percentile category—*n* (%)				
Underweight	9 (10.0%)	5 (10.9%)	4 (9.1%)	0.021
Normal weight	60 (66.7%)	36 (78.3%)	24 (54.5%)	
Overweight/obese	21 (23.3%)	5 (10.9%)	16 (36.4%)	

Normally distributed data presented as mean (SD), and *t*-tests used to compare group differences. Skewed data presented as median (IQR), and Mann–Whitney tests used to compare group differences. Categorical data presented with frequencies (*n*, %), and chi-square tests used to examine distribution differences. BMI: body mass index; BIA: bioelectrical impedance analysis.

**Table 2:** Eating habits between adolescent dancers and non-dancers *.

Eating Habits	Total (*n* = 90)	Dancers Group (*n* = 46)	Controls Group (*n* = 44)	*p*
More than 3 meals/snacks per day	30 (33.3%)	17 (37.0%)	13 (29.5%)	0.507
Daily breakfast consumption	61 (67.8%)	33 (71.7%)	28 (63.6%)	0.500
Daily fruit consumption	51 (56.7%)	30 (65.2%)	21 (47.7%)	0.136
Daily vegetable consumption	33 (36.7%)	21 (45.7%)	12 (27.3%)	0.083
Daily dairy consumption	57 (63.3%)	31 (67.4%)	26 (59.1%)	0.513
Daily cereal or bread consumption	45 (50.0%)	24 (52.2%)	21 (47.7%)	0.833
Daily olive oil consumption	28 (31.1%)	16 (34.8%)	12 (27.3%)	0.499
Consumption of fish ≥ twice/week	14 (15.6%)	9 (19.6%)	5 (11.4%)	0.386
Consumption of nuts ≥ twice/week	18 (20.0%)	12 (26.1%)	6 (13.6%)	0.189
Consumption of eggs ≥ twice/week	25 (27.8%)	11 (23.9%)	14 (31.8%)	0.483
Consumption of legumes ≥ twice/week	38 (42.2%)	19 (41.3%)	19 (43.2%)	1.000
Consumption of red meat ≤ twice/week	47 (52.2%)	24 (52.2%)	23 (52.3%)	1.000
Consumption of poultry ≤ twice/week	55 (61.1%)	32 (69.6%)	23 (52.3%)	0.130
Consumption of soft drinks ≤ twice/week	52 (57.8%)	29 (63.0%)	23 (52.3%)	0.394
Consumption of low-fat products	45 (50.0%)	21 (45.7%)	24 (54.5%)	0.527
Consumption of no sugar products	40 (44.4%)	18 (39.1%)	22 (50.0%)	0.396
Being vegetarian	12 (13.5%)	8 (17.8%)	4 (9.1%)	0.353
Use of nutritional supplements	12 (13.3%)	9 (19.6%)	3 (6.8%)	0.120

* Categorical data presented with frequencies (*n*, %), and chi-square tests used to examine distribution differences.

**Table 3:** BMI and fat mass (mean and SD) according to eating habits *.

Eating Habits		*n*	BMI (kg/m^2^)	*p*	Fat Mass (kg)	*p*
More than 3 eating occasions/day	Yes	30	19.9 (2.8)	0.028	11.7 (4.6)	0.069
	No	60	21.7 (3.9)		14.3 (7.0)	
Daily breakfast consumption	Yes	61	20.5 (3.2)	0.042	12.4 (5.7)	0.020
	No	29	22.4 (4.3)		15.7 (7.2)	
Daily fruit consumption	Yes	51	21.1 (3.6)	0.875	13.5 (6.0)	0.986
	No	39	21.2 (3.7)		13.5 (6.9)	
Daily vegetable consumption	Yes	33	21.8 (3.9)	0.157	14.3 (6.9)	0.329
	No	57	20.7 (3.5)		13.0 (6.1)	
Daily dairy consumption	Yes	57	21.4 (3.8)	0.257	14.0 (6.6)	0.345
	No	33	20.5 (3.3)		12.6 (6.0)	
Daily cereal or bread consumption	Yes	45	20.5 (3.3)	0.148	12.7 (5.5)	0.258
	No	45	21.7 (3.9)		14.2 (7.1)	
Daily olive oil consumption	Yes	28	22.1 (4.2)	0.106	14.7 (7.2)	0.237
	No	62	20.7 (3.3)		12.9 (6.0)	
Consumption of fish at least twice/week	Yes	14	21.0 (4.5)	0.878	13.0 (6.9)	0.792
	No	76	21.1 (3.5)		13.5 (6.3)	
Consumption of nuts at least twice/week	Yes	18	18.6 (2.4)	0.001	9.1 (3.5)	0.001
	No	72	21.7 (3.7)		14.6 (6.5)	
Consumption of eggs at least twice/week	Yes	25	20.5 (3.9)	0.336	12.3 (6.0)	0.278
	No	65	21.3 (3.6)		13.9 (6.5)	
Consumption of legumes at least twice/week	Yes	38	21.1 (4.1)	0.900	13.3 (6.4)	0.829
	No	52	21.2 (3.3)		13.6 (6.4)	
Consumption of red meat less than twice/week	Yes	47	21.3 (3.6)	0.600	14.0 (6.0)	0.404
	No	43	20.9 (3.8)		12.9 (6.8)	
Consumption of poultry less than twice/week	Yes	55	20.9 (3.0)	0.436	13.0 (5.5)	0.378
	No	35	21.5 (4.5)		14.2 (7.6)	
Consumption of soft drinks less than twice/week	Yes	52	21.2 (3.6)	0.822	13.8 (6.2)	0.570
	No	38	21.0 (3.8)		13.0 (6.7)	

* Independent t-tests used to compare differences between groups.

**Table 4:** Participants’ scores in EAT-26 (median and IQR) and count of participants that scored ≥ 20.

EAT-26 Scores	Total (*n* = 90)	Dancers Group (*n* = 46)	Control Group (*n* = 44)	*p*
Total ΕAΤ-26 score ≥ 20—*n* (%)	21 (23.3%)	11 (23.9%)	10 (22.7%)	0.894
Total score	11.0 (8.0, 19.0)	10.5 (7.0, 19,0)	12.0 (8.5, 18.5)	0.518
Dieting scale	6.0 (3.0, 10.0)	5.0 (3.0, 13.0)	7.0 (4.0, 10.0)	0.207
Bulimia scale	0.0 (0.0, 2.0)	0.0 (0.0, 1.0)	1.0 (0.0, 3.0)	0.085
Oral Control scale	4.0 (2.0, 7.0)	5.0 (2.0, 8.0)	4.0 (2.0, 6.0)	0.171

Mann–Whitney tests used to compare group differences in EAT-26 scores. Categorical data presented with frequencies (*n*, %), and chi-square tests used to examine distribution differences.

**Table 5:** Participants’ anthropometric characteristics according to the EAT-26 score.

	ΕAΤ-26 ≥ 20 (*n* = 21)	ΕAΤ-26 < 20 (*n* = 69)	*p*
Age (years)	13.4 (1.2)	13.8 (1.3)	0.233
Height (cm)	160.5 (6.4)	160.9 (5.9)	0.820
Body weight (kg)	59.0 (11.1)	53.5 (9.9)	0.032
BMI (kg/m^2^)	22.8 (4.0)	20.6 (3.4)	0.014
Waist circumference (cm)	71.0 (68.0, 75.0)	68.0 (64.0, 73.0)	0.022
Hips circumference (cm)	98.4 (9.1)	93.4 (8.4)	0.023
Fat mass (kg)—Skinfolds	16.1 (6.5)	12.7 (6.2)	0.033
Fat mass (kg)—BIA	16.0 (7.0)	13.9 (6.1)	0.201
Fat mass (%)—Skinfolds	26.3 (6.4)	22.7 (7.2)	0.042
Fat mass (%)—BIA	26.2 (7.0)	25.3 (6.9)	0.606
Fat free mass (kg)—BIA	42.9 (5.9)	39.3 (4.9)	0.008

Normally distributed data presented as mean (SD), and t-tests used to compare group differences. Skewed data presented as median (IQR), and Mann–Whitney tests used to compare group differences. BMI: body mass index; BIA: bioelectrical impedance analysis.

**Table 6:** DEBs and body dissatisfaction characteristics.

DEBs and Body Dissatisfaction Characteristics	Total (*n* = 90)	Dancers Group (*n* = 46)	Control Group (*n* = 44)	*p*
Dieting at the moment	10 (11.2%)	5 (11.1%)	5 (11.4%)	>0.999
Ever on a diet	32 (35.6%)	15 (32.6%)	17 (38.6%)	0.661
Ever followed a diet that was not prescribed by health expert	28 (31.1%)	16 (34.8%)	12 (27.3%)	0.499
Ever used vomiting/laxatives	3 (3.3%)	3 (6.5%)	0 (0.0%)	0.242
Weighing herself once a week or more frequently	26 (28.9%)	14 (30.4%)	12 (27.3%)	0.818
Not satisfied with her body	42 (47.7%)	20 (44.4%)	22 (51.2%)	0.670
Feeling normal but wanting to weigh less	21 (24.1%)	11 (25.6%)	10 (22.7%)	0.403
Has lost weight in order to participate in dancing exams or dancing performance		8 (17.4%)		
Feeling that her body is not ideal for dancer		18 (39.1%)		
Received diet advice from coaches		6 (13.0%)		
Received diet advice from teammates		4 (8.7%)		
Received diet advice from parents		12 (26.1%)		

Data presented with frequencies (*n*, %), and chi-square test used to examine distribution differences.

## Data Availability

All data are available upon request to the first author.
